# Effect of quantitative values on shortened acquisition duration in brain tumor ^11^C-methionine PET/CT

**DOI:** 10.1186/s40658-021-00379-2

**Published:** 2021-03-31

**Authors:** Masatoshi Morimoto, Nobuyuki Kudomi, Yukito Maeda, Takuya Kobata, Akihiro Oishi, Keisuke Matsumoto, Toshihide Monden, Takanobu Iwasaki, Katsuya Mitamura, Takashi Norikane, Yuka Yamamoto, Yoshihiro Nishiyama

**Affiliations:** 1 Division of Social and Environmental Medicine, Graduate School of Medicine, Kagawa University, 1750-1 Ikenobe, Miki-cho, Kita-gun, Kagawa 761-0793 Japan; 2 Department of Clinical Radiology, Kagawa University Hospital, Kita-gun, Kagawa 761-0793 Japan; 3 Department of Medical Physics, Faculty of Medicine, Kagawa University, Kita-gun, Kagawa 761-0793 Japan; 4 Faculty of Health and Welfare, Tokushima Bunri University, 1314-1 Shido, Sanuki-city, Kagawa 769-2193 Japan; 5 Department of Radiology, Faculty of Medicine, Kagawa University, Kita-gun, Kagawa 761-0793 Japan

**Keywords:** ^11^C-methionine, Acquisition duration, Statistical noise, SUVpeak

## Abstract

**Background:**

The amount of signal decreases when the acquisition duration is shortened. However, it is not clear how this affects the quantitative values. This study aims to clarify the effect of acquisition time shortening in brain tumor PET/CT using ^11^C-methionine on the quantitative values.

**Method:**

This study was a retrospective analysis of 30 patients who underwent clinical ^11^C-methionine PET/CT examination. PET images were acquired in list mode for 10 min. PET images of acquisition duration from 1 to 10 min with 1-min step were reconstructed. We examined the effect on the quantitative values of acquisition duration. We placed a volume of interest to include the entire tumor and regions of interest in the shape of a large crescent in the contralateral hemisphere in 5 contiguous axial slices as normal tissue. Quantitative values examined were maximum, peak, and mean standardized uptake values (SUVmax, SUVpeak, SUVmean), metabolic tumor volume (MTV), and maximum tumor to normal tissue ratio (TNRmax), with each duration compared to that with 10 min.

**Results:**

SUVmax, MTV, and TNRmax showed the highest values due to the effects of statistical noise when the acquisition time was 1 min. These values were stable when the acquisition duration was > 6 min. SUVpeak and SUVmean showed mostly consistent values regardless of duration.

**Conclusions:**

SUVmax, MTV, and TNRmax are affected by acquisition time. If the acquisition duration was > 6 min, the fluctuation could be suppressed within 5% in these quantitative values. However, SUVpeak was suggested to be a robust index regardless of the acquisition duration.

**Supplementary Information:**

The online version contains supplementary material available at 10.1186/s40658-021-00379-2.

## Introduction

Positron emission tomography/computed tomography (PET/CT) with administration of ^11^C-methionine can be used for purposes such as discriminating between radiation necrosis and recurrence after radiation therapy, between tumor and non-tumor lesions, and for determination of the extent of brain tumor invasion. ^11^C-methionine PET/CT has been reported to be more useful than CT, magnetic resonance imaging (MRI), or ^18^F-fluoro-2-deoxy-D-glucose ([^18^F]FDG)-PET/CT [1–3].

Currently, some clinical indexes such as standardized uptake value (SUV), maximum SUV within a region of interest (SUVmax) [4–6], peak SUV (SUVpeak) [7, 8], metabolic tumor volume (MTV) [9], and maximum tumor to normal tissue ratio (TNRmax) [10, 11] are widely used for diagnostic purposes in ^11^C-methionine PET/CT.

To obtain these indexes with ^11^C-methionine PET/CT, an appropriate acquisition time has been reported to be 10 min, according to the standard protocol established by the Japanese Society of Nuclear Medicine (JSNM) [12]. But even with a short duration of 10 min, some patients with conditions such as brain tumor cannot always remain motionless in the gantry. Some methods are available for correcting positional deviation such as respiratory synchronization in the trunk [13, 14] or setting the CT scanning time at a low speed so as to match it with that of PET [15]. However, these methods are limited for correcting body movements caused by breathing and do not compensate for sudden body movements. One possible solution would be to fix the patient tightly on the gantry, but, if the fixation is too tight, it would be uncomfortable or even painful for most patients, thereby possibly inducing further movements. An alternative solution would be reexamination, but the half-life of ^11^C is as short as 20.4 min, and a sufficient statistical amount of signal cannot be obtained due to reduced radioactivity. In addition, the burden to the patient would be appreciably increased. Furthermore, in the practical clinical setting, multiple patients are often examined in 1 day, and an additional inclusion of an examination for such a patient would induce delay of the following examination start times for waiting patients and therefore reduction of the radioactivity to be administered. Thus, in general, reexamination is not practical.

Recent PET scannings are performed in list mode, and images can be obtained by extracting an arbitrary part of the acquisition sinogram data. In other words, it is possible to use the data up to just before any movement. However, since the acquisition time becomes less than 10 min, drawbacks such as decreases in the amount of signal and changes in the quantitative values in the reconstructed image are induced. Ensuring accuracy of the above clinical indexes is important for the diagnosis. However, there are, so far, no reports on the effect on the image, namely, to what extent the quantitative accuracy is adversely affected in ^11^C-methionine examination. In this study, we tested the effects on quantitative values of those clinical indexes on ^11^C-methionine PET/CT when the acquisition duration is shortened.

## Materials and methods

### Data acquisition and image reconstruction

A Biograph mCT64-4R scanner (Siemens Healthcare) was used for all PET acquisitions in this study. The PET data were acquired in 3-dimensional mode for 10 min in list mode. They were reconstructed using the ordered subsets expectation maximization (OSEM) algorithm with the point spread function (PSF) correction and time-of-flight (TOF) technique. The image matrix was 256 × 256, with 1.27-mm pixels. The reconstruction parameters for OSEM + PSF + TOF were 5 iterations and 21 subsets. The PET image slice thickness was 3 mm. A Gaussian filter with a full width at half maximum (FWHM) of 3 mm was used as a post-smoothing filter. Scanning parameters for CT were as follows: 120 kV, 28 mA, 3-mm slice thickness, and 1.0-s rotation. The CT data were used for the attenuation correction.

### Clinical study

We retrospectively collected clinical data of ^11^C-methionine PET/CT for examining brain tumors. A total of 30 patients (14 males and 16 females) who underwent ^11^C-methionine PET/CT between September 6, 2017, and March 31, 2019, were analyzed (Supplementary material, Table S1). The mean age was 61.0 ± 15.4 years (range 34–85 years). The patients had an average weight of 58.9 ± 10.8 kg (range 44.3–76.9 kg). No pretreatment such as fasting was done. This clinical study was approved by the ethics committee of our institution (Heisei 30-021).

Patients were injected intravenously with 346.3 ± 54.9 MBq (range, 254.3–451.4 MBq) of ^11^C-methionine. The injection dose per kilogram averaged 5.95 ± 0.73 MBq/kg (range, 4.97–6.95 MBq/kg). Acquisition of PET images began at 18.45 ± 3.22 min (range, 14.50–27.35 min) after injection.

### Data analysis

**Fig. 1:**
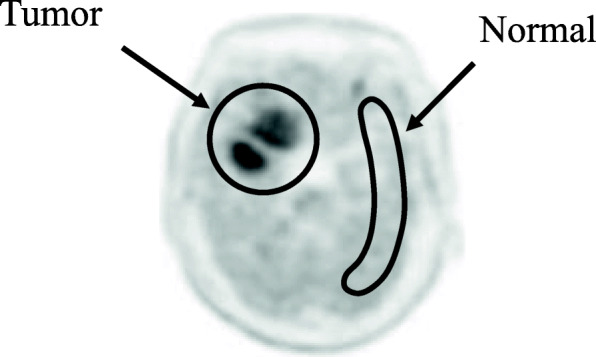
VOI and ROI setting in clinical image

PET image reconstruction was performed extracting the list mode data from 1 to 10 min duration in 1-min steps to determine the effect of shortening the acquisition duration. We placed the volume of interest (VOI) to include the entire tumor and a region of interest (ROI) in the shape of a large crescent in the contralateral hemisphere to the tumor as normal tissue (Fig. 1). The ROI of normal tissue was placed in 5 contiguous axial slices, and the mean SUV (SUVmean) was calculated. For data analysis, Syngo.via client 3.0 (Siemens Healthcare) was used. Evaluation of quantitative values in the clinical images was performed by analyzing the relationship between acquisition duration and SUVmax, SUVpeak, SUVmean, MTV, and TNRmax. SUVmax, SUVpeak, SUVmean, and MTV were calculated for the VOI placed. It was an automatic search of the SUVpeak within the tumor VOI, meaning that the positions of the pixels for the SUVpeak were not always identical among the images with different acquisition durations. The MTV was defined as the sum of voxel volumes in the placed VOI that measured the SUV of > 1.3-fold to the SUVmean in normal tissue [3, 9, 16, 17].

The TNRmax was calculated as shown below, by dividing the SUVmax of the tumor by the SUVmean of the normal tissue. Mean ratio of %difference in the SUVmax, SUVpeak, MTV, and TNRmax were calculated as the difference between the quantitative value for each duration and the quantitative value for the acquisition time of 10 min divided by the quantitative value for the acquisition time of 10 min, multiplied by 100.

$$ \mathrm{TNRmax}=\frac{\mathrm{SUVmax}\ \left(\mathrm{tumor}\right)}{\mathrm{SUVmean}\ \left(\mathrm{normal}\ \mathrm{tissue}\right)} $$

## Results

**Fig. 2:**
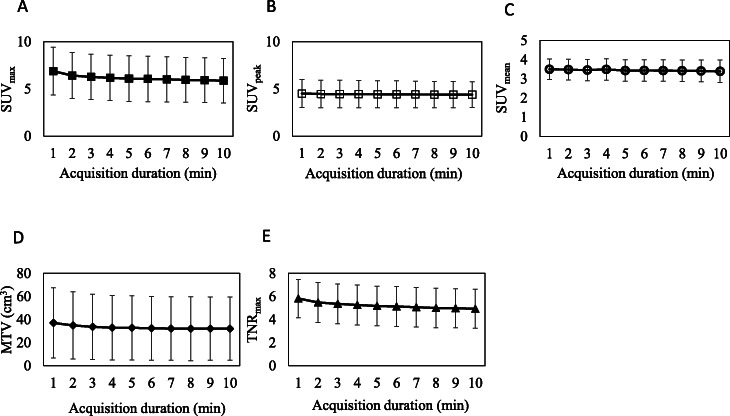
Change of SUVmax (**a**), SUVpeak (**b**), SUVmean (**c**), MTV (**d**), and TNRmax (**e**) (*n* = 30). Error bars indicate SD

Figure 2 shows the relationships between acquisition time and each clinical index.

Figure 2a and Table 1 show the relationship between acquisition duration and SUVmax as well as the mean ratio of change. As the duration shortened, SUVmax values became gradually higher and at 1-min duration were the highest, namely 5.87 for 10 min and 6.89 for 1min (Fig. 2a). When the duration was > 5 min, SUVmax values were acceptably small with a < 5% increase (Table 1).

**Table 1:** Mean ratio of %difference in SUVmax, SUVpeak, SUVmean, MTV, and TNRmax for each duration against a 10-min acquisition time

Duration (min)	1	2	3	4	5	6	7	8	9
ΔSUVmax	+ 19.6 ± 12.1	+ 10.8 ± 8.7	+ 8.0 ± 7.9	+ 6.0 ± 5.9	+ 4.2 ± 4.0	+ 3.6 ± 3.2	+ 2.8 ± 2.4	+ 1.7 ± 1.8	+ 1.1 ± 1.1
ΔSUVpeak	+ 3.1 ± 3.6	+ 1.9 ± 3.3	+ 1.5 ± 2.6	+ 1.3 ± 2.3	− 1.0 ± 1.9	+ 0.8 ± 1.6	+ 0.6 ± 1.1	+ 0.3 ± 0.9	+ 0.1 ± 0.5
ΔSUVmean	+ 3.6 ± 6.2	+ 3.2 ± 5.4	+ 2.2 ± 3.8	+ 3.0 ± 7.4	− 1.5 ± 3.5	+ 1.6 ± 3.5	+ 1.4 ± 3.4	+ 1.1 ± 3.2	+ 0.9 ± 3.1
ΔMTV (cm^3^)	+ 19.8 ± 16.7	+ 11.5 ± 10.8	+ 6.8 ± 10.1	+ 4.1 ± 8.1	+ 3.4 ± 6.1	+ 1.4 ± 4.1	+ 0.7 ± 3.1	− 1.1 ± 7.6	+ 0.4 ± 2.4
ΔTNRmax	+ 21.3 ± 16.5	+ 12.6 ± 10.1	+ 9.9 ± 8.9	+ 7.4 ± 7.2	+ 5.4 ± 5.1	+ 4.3 ± 4.5	+ 2.8 ± 3.3	+ 1.5 ± 1.9	+ 0.9 ± 1.2

Figure 2b and Table 1 show the relationship between acquisition duration and SUVpeak and the mean ratio of change. SUVpeak values were mostly consistent independent of the duration.

Figure 2c and Table 1 show the relationship between acquisition duration and SUVmean and the mean ratio of change. SUVmean values were also mostly consistent independent of the duration.

Figure 2d and Table 1 show the relationship between acquisition duration and MTV and the mean ratio of change. As the duration shortened, MTV values became gradually higher and at 1-min duration were the highest, namely 32.1 cm^3^ for 10 min and 37.1 cm^3^ for 1 min (Fig. 2d). When the duration was > 4 min, the MTV values were acceptably small with a < 5% increase (Table 1).

Figure 2e and Table 1 show the relationship between acquisition duration and TNRmax and the mean ratio of change. As the duration shortened, TNRmax values became gradually higher and at 1-min duration were the highest, namely 4.94 for 10 min and 5.82 for 1 min (Fig. 2e). When the duration was > 6 min, TNRmax values were acceptably small with a < 5% increase (Table 1).

**Fig. 3:**
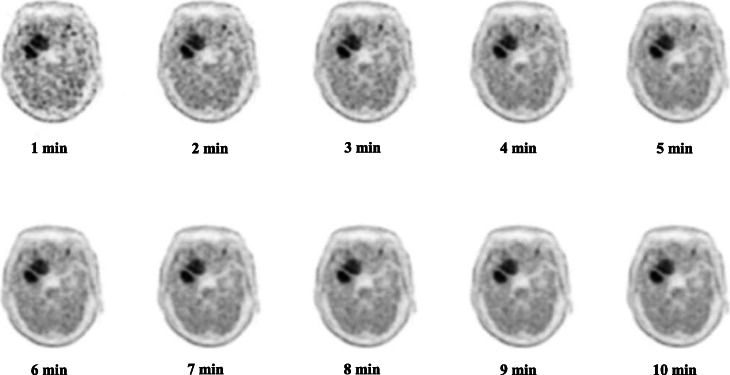
The images with acquisition duration from 1 to 10 min

Figure 3 illustrates the representative images with acquisition duration from 1 to 10 min.

## Discussion

In this study, we tested the influence of shortening the acquisition duration on the quantitative values in ^11^C-methionine PET study. We found that the quantitative values changed with the change of the acquisition duration. In a conventional procedure limited to viewing images on a display, it is not clear how the quantitative values change when the acquisition duration is shortened. The present results revealed that the quantitative values changed as the acquisition duration was shortened.

SUVmax increased when the acquisition time was shortened. The noise increases as the acquisition time shortens in clinical images. As the noise increases, the SUVmax in the high integrated region increases by fluctuation [18]. Therefore, we considered that the quantitative values changed due to the influence of noise when the acquisition time shortened.

SUVpeak was mostly consistent regardless of the acquisition duration. Akamatsu et al. demonstrated that SUVpeak is a highly reproducible index not easily affected by statistical fluctuation [19], because SUVpeak is obtained by the average value within 1 cm^3^ area with metabolic accumulation being most active. Therefore, the influence of statistical noise was suppressed, and the index value did not change so much.

The quantitative value of MTV was stable when the duration was > 4 min. Uptake of ^11^C-methionine was less in the normal tissue, and there was less fluctuation independent of acquisition duration. Since the threshold was set as 1.3-fold of the SUVmean of normal tissue, that did not change so much independent of the acquisition duration. On the other hand, in tumors, the degree of uptake was high and the concentration and therefore SUV were also high, and so the fluctuation became large, and consequently, the area exceeding the threshold increased. Thus, the MTV increased when duration was < 4 min. In studies using FDG, the threshold is set at an SUV value of 2.5 or 40% of SUVmax [20, 21], and Lim et al. reported that MTV may vary depending on the threshold [22]. Their finding suggests that MTV in ^11^C-methionine also varies depending on the threshold setting, and different trends could be found other than those recognized in this study.

TNRmax is obtained by SUVmax divided by SUVmean. SUVmean was less dependent on the acquisition duration and mostly consistent as noted above. Therefore, TNRmax showed similar dependency on scan time to SUVmax.

^11^C-methionine PET is often evaluated using SUVmax as an index [18, 23]. However, as has been shown, SUVmax varied greatly depending on the acquisition time. Lodge et al. reported that, for images with larger noise degree, SUVpeak is a more robust index than SUVmax in [^18^F]FDG study [6]. Akamatsu et al. [19] also reported that it is desirable to use SUVpeak, which is less affected by image noise, when accurate quantification is required. The present study with ^11^C-methionine also showed that SUVpeak with less dependency on acquisition time would be a robust index for diagnosis. Therefore, if shortening the acquisition time is unavoidable, SUVpeak would be more practical for evaluation than SUVmax. It should be noted that SUVpeak requires a VOI size of 12 mm or more in diameter and cannot be used for smaller lesions.

According to the European Association of Nuclear Medicine (EANM), the Society of Nuclear Medicine and Molecular Imaging (SNMMI), the European Association of Neurooncology (EANO), and the working group for Response Assessment in Neurooncology with PET (PET-RANO), it is recommended to start acquisition 10 min after injection and scan for 20 min [24]. The standards differ between EANM/EANO/RANO/SNMMI and our JSNM, and our procedure with 10 min duration and around 20 min after injection is different. The amount of signal in our procedure is expected to be smaller compared to that in the EANM/EANO/RANO/SNMMI guidelines. However, when as the present study showed the acquisition duration was more than 6 min, the change in the quantitative values for all indexes was acceptably small in this study. This would suggest that our findings can be applied to the indexes from images acquired with the EANM/EANO/RANO/SNMMI guidelines.

Our salient finding in the present study was the change of clinical indexes when the duration was shortened due to noise, and consequently more than 6 min duration was required. Currently, dynamic and texture analyses are proposed as the evaluation for brain tumor images with amino acid tracers [25, 26]. Dynamic analysis would predict the molecular features of the tumor but requires around 30-min acquisition time [25]. Textural analysis would be applied for predicting the isocitrate dehydrogenase genotype, but image quality has the biggest influence on the results of textural feature analysis [26]. Therefore, shortening the acquisition time is not acceptable for these methods. An alternative is to apply denoise or increase the number of counts technique with artificial intelligence [27–29]. This requires huge amount of cases but is not practical for our data set with only 30 cases.

This study had some limitations. First, we did not evaluate the detectability of lesions, which from a diagnostic aspect is important. Further studies are required to evaluate the detectability of lesions. Second, we used OSEM + PSF + TOF for the reconstruction algorithm. No other reconstruction algorithm was used in this study. PSF correction causes a Gibbs artifact and increases the risk of noise [30]. Quantitative values are expected to vary when other reconstruction algorithms without PSF correction are used. Third, the PET/CT we used is a photomultiplier tube (PMT)-based detector. Recently, silicon photomultiplier tube (SiPM)-based detectors have emerged and have higher sensitivity and timing resolution than PMT. Quantitative values are expected to be different when using PET/CT with SiPM-based detectors.

## Conclusion

In this study, we tested the influence of shortening the acquisition duration on the quantitative value in ^11^C-methionine PET study. We revealed the degree of change in clinical indexes with shortening of the acquisition duration. These changes cannot be quantitatively estimated only by viewing images.

The SUVmax, MTV, and TNRmax were not stable due to the influence of statistical noise when the acquisition duration was shortened. Acquisition duration required 6 min within 5% error in these quantitative values. It should be noted that the quantitative values have an error of > 5% when using the images of < 6 min for unavoidable reasons such as body movements. SUVpeak is a highly reproducible index not easily affected by acquisition duration.

## Supplementary Information

**Additional file 1: Supplementary material**, **Table S1**. Individual patient data

## Data Availability

The data that support the findings of this study are available from the Ethics Committee of Kagawa University Hospital, but restrictions apply to the availability of these data, which were used under license for the current study, and so are not publicly available. Data are however available from the authors upon reasonable request and with permission of the Ethics Committee of Kagawa University Hospital.

## Abbreviations

PET/CT: Positron emission tomography/computed tomography
MRI: Magnetic Resonance Imaging
[^18^F]FDG: ^18^F-fluoro-2-deoxy-D-glucose
SUV: Standardized uptake value
MTV: Metabolic tumor volume
T/M ratio: Tumor to normal tissue ratio
OSEM: Ordered subsets expectation maximization
PSF: Point spread function
TOF: Time-of-flight
FWHM: Full width at half maximum
VOI: Volume of interest
ROI: Region of interest
PMT: Photomultiplier tube
SiPM: Silicon photomultiplier tube
