# Supplementary material for: Effect of quantitative values on shortened acquisition duration in brain tumor 11C-methionine PET/CT
Source: EJNMMI Phys. 2021 Mar 31;8:34. doi: 10.1186/s40658-021-00379-2 (PMC8012475; doi:10.1186/s40658-021-00379-2)
Supplement: Supplementary file 1 — Additional file 1: Supplementary material, Table S1. Individual patient data [file 40658_2021_379_MOESM1_ESM.docx]

| Patient ID | Age (y) | Diagnosis | Localization | WHO grade | Newly diagnosed (ND) /  recurrence (REC) |
| --- | --- | --- | --- | --- | --- |
| pat1 | 84 | meningioma | Left tempral | 1 | ND |
| pat2 | 79 | meningioma | Right frontal | - | ND |
| pat3 | 75 | glioblastoma | Right frontal, temporal | 4 | ND |
| pat4 | 35 | anaplastic astrocytoma | Left temporal | 3 | ND |
| pat5 | 75 | glioblastoma | Right frontal | 4 | ND |
| pat6 | 59 | glioblastoma | Right thalamus | 4 | ND |
| pat7 | 34 | glioblastoma | Right frontal | 4 | REC |
| pat8 | 68 | meningioma | Right falx cerebri | 2 | ND |
| pat9 | 59 | meningioma | Left middle cranial fossa | 1 | REC |
| pat10 | 69 | meningioma | Right parasaggital | 1 | ND |
| pat11 | 79 | glioblastoma | Left parietal | 4 | ND |
| pat12 | 63 | glioblastoma | Left occipital | 4 | ND |
| pat13 | 41 | oligodendroglioma | Left occipital | 2 | ND |
| pat14 | 50 | meningioma | Right parasaggital | - | ND |
| pat15 | 53 | meningioma | Left frontal | 2 | ND |
| pat16 | 56 | glioblastoma | Left parietal | 4 | ND |
| pat17 | 70 | meningioma | Left frontal | 2 | ND |
| pat18 | 38 | oligodendroglioma | Left frontal | 2 | ND |
| pat19 | 45 | meningioma | Right falx cerebri | - | ND |
| pat20 | 63 | glioblastoma | Right frontal | 4 | ND |
| pat21 | 65 | Ependymoma | Left lateral ventricle | 2 | ND |
| pat22 | 56 | glioblastoma | Left parietal | 4 | ND |
| pat23 | 37 | anaplastic astrocytoma | Right frontal | 3 | REC |
| pat24 | 79 | meningioma | Left falx cerebri | 2 | ND |
| pat25 | 74 | meningioma | Left lateral ventricle | - | ND |
| pat26 | 69 | metastasis | Right occipital | - | REC |
| pat27 | 36 | N/A | Left occipital | - | ND |
| pat28 | 60 | glioblastoma | Right temporal | 4 | ND |
| pat29 | 85 | glioblastoma | Left temporal, occipital | 4 | ND |
| pat30 | 74 | CNS lymphoma | Left thalamus | - | ND |

Supplemental Information

Table S1 Individual patient data

Abbreviations

N/A: Not Applicable; CNS: central nervous system
